# The optic nerve head in glaucoma

**Published:** 2022-01-31

**Authors:** Rupert RA Bourne, Tasneem Khatib

**Affiliations:** 1 Consultant Ophthalmic Surgeon and glaucoma specialist: Cambridge University Hospitals, UK, and Professor of Ophthalmology, Vision and Eye Research Institute, Anglia Ruskin University, UK.; 2 Glaucoma Fellow, Cambridge University Hospitals, UK.

**The key to detection and management of glaucoma is understanding how to examine the optic nerve head.**

All types of glaucoma involve glaucomatous optic neuropathy. The key to detection and management of glaucoma is understanding how to examine the optic nerve head (ONH).

This article addresses the following issues:

- How to examine the ONH
- Normal characteristics of the ONH
- Characteristics of a glaucomatous ONH
- How to tell if the glaucomatous optic neuropathy is getting worse.

**Figure 1:**
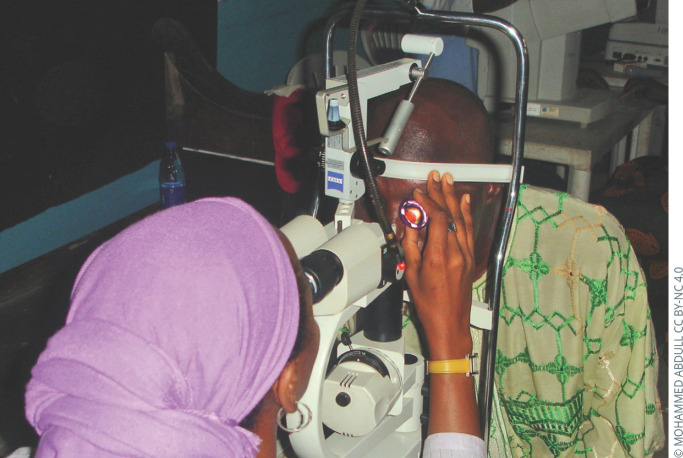
Examining the optic nerve using a slit lamp and posterior pole lens. This offers a stereo view, high magnification, and good illumination and is best performed through a dilated pupil.

The ONH can be examined using a direct ophthalmoscope, an indirect ophthalmoscope, or a posterior pole lens with a slit lamp (Figure 1).

Many types of health professional can assess the ONH accurately after having appropriate training. Dilating the pupil makes this easier and will improve the accuracy of the examination, regardless of which instrument is used. Where the equipment is available, more sophisticated techniques such as ocular coherence tomography can also be used to complement the clinical examination of the ONH and provide quantitative measurements.

The time available to view the ONH is often short as the examination is uncomfortable for the patient. It is therefore essential that the examiner has a strategy for making the observations needed to distinguish a glaucomatous ONH from a normal ONH.

Before you start, you should first be able to recognise the characteristics of both a normal and a glaucomatous ONH, and be able to look for additional signs that could indicate a glaucomatous ONH.

**Figure 2:**
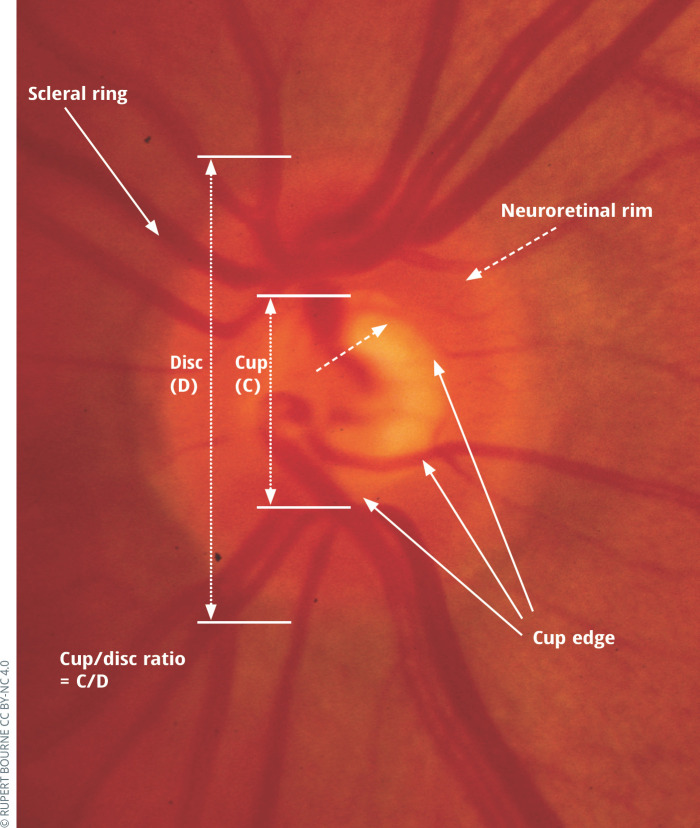
Normal optic nerve head.

**Characteristics of the normal ONH (Figure 2)**

The ONH, or optic disc, is a round/oval ‘plughole’ down which more than a million retinal nerve fibres descend through a sieve-like sheet known as the lamina cribrosa. The retinal nerve fibres are then bundled together behind the eye to form the optic nerve which then continues towards the brain.

**Figure 3:**
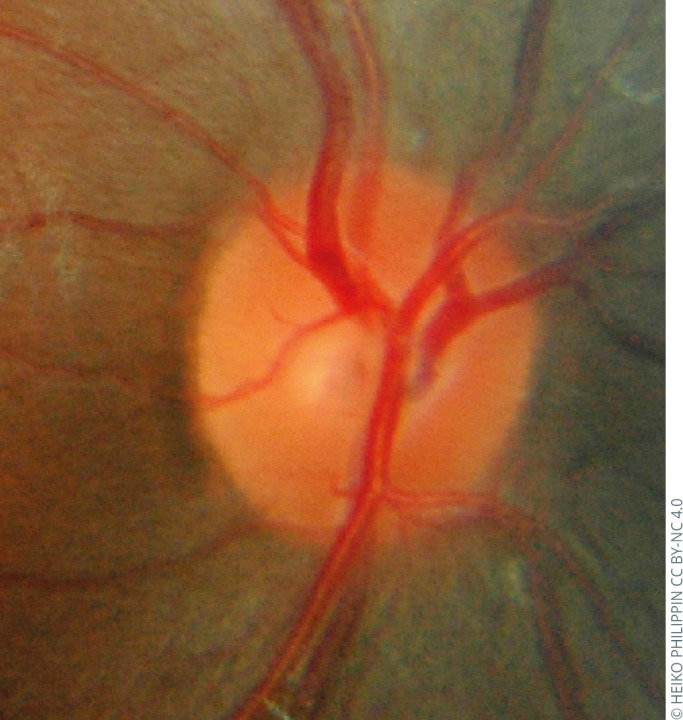
Normal optic nerve head of a young African patient.

The retinal nerve fibres are spread unevenly across the surface of the retina in a thin layer which has a ‘feathery’ appearance, best seen immediately above and below the disc (Figure 3).

As the nerve fibres approach the edge of the disc they pour over the scleral ring (which marks the edge of the disc) and then down its inner surface. The dense packing of nerve fibres just inside the scleral ring is visualised as the neuroretinal rim. The cup is the area central to the neuroretinal rim. The cup edge (where it meets the neuroretinal rim) is best seen by the bend in small and medium-sized blood vessels as they leave, or descend into, the cup.

Most normal discs are more vertically oval and their cup more horizontally oval.

In addition, most (but not all) normal ONHs obey the ‘ISNT’ rule: the Inferior (lower) rim is usually thicker than the Superior (upper) rim, which is thicker than the Nasal rim (inner, nearest the nose). The Temporal rim (outer, nearest the temple) is the thinnest.

**Characteristics of a glaucomatous ONH**

**Figure 4:**
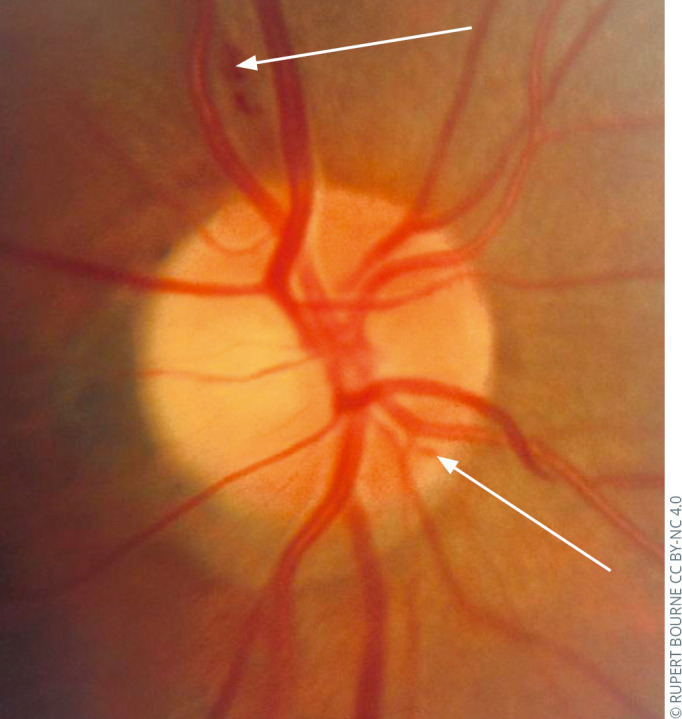
Glaucomatous optic neuropathy: splinter haemorrhages.
- Thinning of the neuroretinal rim (usually at the superior and inferior poles) (e.g., Figures 5 and 6)

- Generalised/focal enlargement of the cup. (Note that the cup always appears smaller when viewed monoscopically than in stereo)
- Disc haemorrhage (within one disc diameter of ONH) (Figure 4)

**Figure 5:**
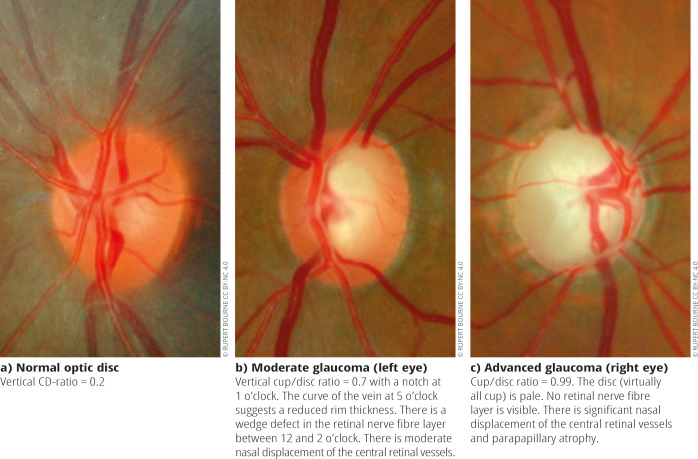
Normal optic disc (a) and glaucomatous optic nerve heads of two patients with different severities of glaucoma.

**Figure 6:**
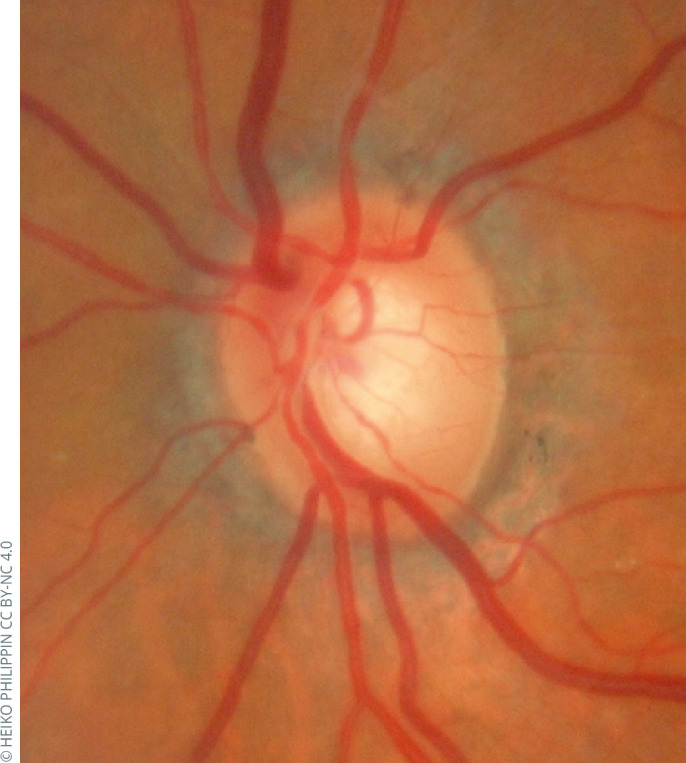
Glaucomatous optic nerve head of a patient with pseudoexfoliation glaucoma (PXFG). The demarcation of the cup by the blood vessels differs from the margin between the pallor of the base of the cup and the surrounding pinker colour between this and the disc edge. Focussing on the colour difference is misleading. One should judge the edge of the rim by the change in direction of the small and medium-sized vessels which, in this case, indicates a thinner rim than might be suspected by the colour difference.
- Asymmetry of cupping between patient’s eyes
- Loss of nerve fibre layer (Figure 7).

**Figure 7:**
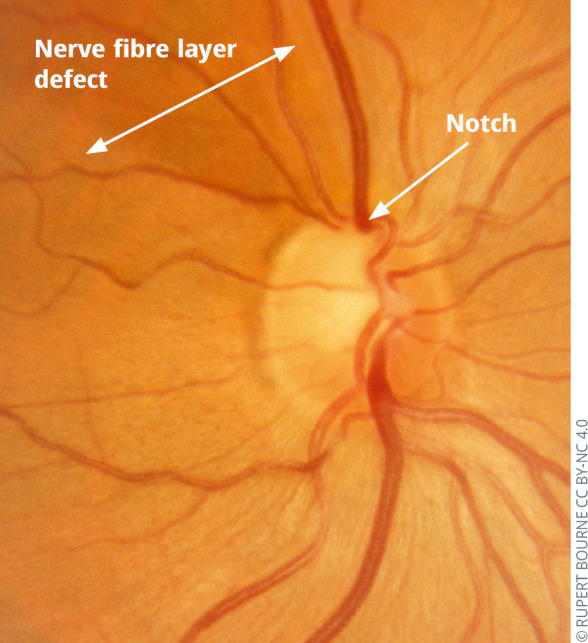
Glaucomatous optic neuropathy: focal enlargement of cup (notch) and nerve fibre layer defect.

**Additional signs which should heighten suspicion of a glaucomatous ONH**

- Cup/disc ratio (CDR) ≥0.7. A measurement of CDR alone is insufficient and may be misleading as small discs will have smaller cups and hence a smaller CDR. It is important, therefore, to document disc size by measuring the vertical height of the disc. In most populations, only 5% of people with no glaucoma will have a CDR of ≥0.7.
- Rim does not obey the ISNT rule
- Presence of parapapillary atrophy (more common in glaucomatous eyes).

**Strategy: distinguishing a glaucomatous ONH from a normal ONH**

1. Dilate pupils, if possible and safe to do so.
2. Identify the disc edge and cup edge, and identify the rim.
3. Does the rim thickness obey the ISNT rule?
4. Is there a haemorrhage?
5. Measure the vertical height of the ONH.^*^
6. Estimate the vertical CDR.
7. Examine the retinal nerve fibre layer (using green light).^*^
8. Draw an annotated diagram of the ONH.

* This may only be possible with a slit lamp and posterior pole lens. Note that different lenses used at the slit lamp may make the disc seem larger or smaller than it really is. For example, with a 90 dioptre lens the image magnification is 0.76 (image appears small), so any measurement needs to be divided by 0.76 to be accurate. With a 78 dioptre lens the image magnification is 0.93 and with a 60 dioptre it is 1.15 (image appears large) and with a 66 dioptre lens it is 1.00 (no correction needed).

**Is the glaucomatous optic neuropathy worsening or progressing?**

The appearance of any of the features of a glaucomatous ONH, or the exacerbation of these features compared to a previous record, is indicative of a progression/worsening of the disease.

Disc haemorrhages may be present for two weeks to three months and are an important prognostic sign of progression. An accurate record requires careful observation and a detailed drawing, and photographic documentation (preferably stereophotography) is highly recommended.

Other imaging devices (see below) assist the clinician in detection of glaucoma when it is unclear from clinical examination whether the optic discs are glaucomatous.

These devices also offer progression analyses to look for structural deterioration, but these are not a surrogate for a detailed clinical examination.

Progressive worsening of the visual fields should correlate with structural changes at the ONH.

**Structural imaging of the optic nerve in glaucoma**

As part of the clinical assessment of the optic nerve in glaucoma, the International Council of Ophthalmology (ICO) in their Guidelines for Glaucoma Care suggest that structural imaging of the optic nerve may provide a useful but not essential adjunct to direct ophthalmoscopy, slit lamp biomicroscopy and fundus photography when evaluating the optic nerve.

**Figure 8:**
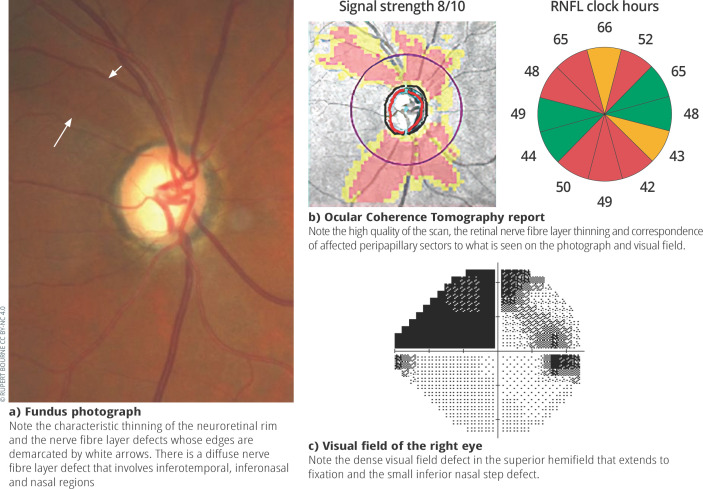
Fundus photograph, ocular coherence tomography report and visual field of the right optic nerve of a patient with glaucoma.

A variety of technologies to analyse the optic nerve are mentioned in the ICO guidelines including confocal scanning laser ophthalmoscopy (Heidelberg Retina Tomograph – HRT), scanning laser polarimetry (GDx Nerve Fibre Analyser – GDx-VCC), and Optical Coherence Tomography (OCT; Figure 8), of which the latter is now the imaging modality of choice. Although structural imaging in isolation using these techniques can differentiate between normal and glaucomatous eyes with mild to moderate visual field loss, none of the current technologies for assessing structural changes of the optic nerve head are suitable for use as an independent screening tool for early to moderate glaucoma or in high risk populations.

**“Image quality is crucial when assessing structural changes, and it can be difficult to distinguish between glaucomatous structural damage and measurement variability or age-related changes.”**

Image quality is crucial when assessing structural changes, and it can be difficult to distinguish between glaucomatous structural damage and measurement variability or age-related changes. Furthermore, each technology has its own optical properties and analysis algorithms and therefore the data acquired is not interchangeable between machines. Monitoring advanced disease can be limited by a floor effect, after which no more thinning is observed. The presence of myopia is also associated with structural thinning of the optic nerve and can confound image interpretation when assessing for glaucomatous progression. These technologies should not replace optic disc photography which remains critically important for the recording of optic disc appearance and subsequent monitoring for change with features such as optic disc haemorrhages not being visible with OCT imaging.

Despite these limitations, structural changes can often precede functional losses and provide an earlier marker of glaucomatous change both in diagnosis and when assessing progression, thus providing useful information during the clinical assessment of the optic nerve in glaucoma.

**Pitfalls and pearls**

- The hallmark of glaucomatous optic neuropathy is excavation of the neuroretinal rim.
- Advanced glaucomatous ONH can result in a pale optic disc, but disc pallor should also raise suspicion of another cause such as optic atrophy.
- A colour difference should not be used to distinguish the cup edge; change in direction of the blood vessels is a more reliable indicator (Figure 2).
- The optic disc abnormality should correlate with the visual field defect. Where this is not the case, further investigations, such as imaging of the brains and orbits with MRI or CT, should be considered.
- The size of the cup always appears smaller when viewed microscopically rather than stereoscopically.
